# B-site ordering and strain-induced phase transition in double-perovskite La_2_NiMnO_6_ films

**DOI:** 10.1038/s41598-018-20812-4

**Published:** 2018-02-06

**Authors:** Sheng-Qiang Wu, Sheng Cheng, Lu Lu, Ming Liu, Xiao-Wei Jin, Shao-Dong Cheng, Shao-Bo Mi

**Affiliations:** 1 State Key Laboratory for Mechanical Behavior of Materials, Xi’an Jiaotong University, Xi’an, 710049 China; 2 School of Microelectronics, Xi’an Jiaotong University, Xi’an, 710049 China

## Abstract

The magnetic and electrical properties of complex oxide thin films are closely related to the phase stability and cation ordering, which demands that we understand the process-structure-property relationships microscopically in functional materials research. Here we study multiferroic thin films of double-perovskite La_2_NiMnO_6_ epitaxially grown on SrTiO_3_, KTaO_3_, LaAlO_3_ and DyScO_3_ substrates by pulsed laser deposition. The effect of epitaxial strains imposed by the substrate on the microstructural properties of La_2_NiMnO_6_ has been systematically investigated by means of advanced electron microscopy. It is found that La_2_NiMnO_6_ films under tensile strain exhibit a monoclinic structure, while under compressive strain the crystal structure of La_2_NiMnO_6_ films is rhombohedral. In addition, by optimizing the film deposition conditions a long-range ordering of B-site cations in La_2_NiMnO_6_ films has been obtained in both monoclinic and rhombohedral phases. Our results not only provide a strategy for tailoring phase stability by strain engineering, but also shed light on tuning B-site ordering by controlling film growth temperature in double-perovskite La_2_NiMnO_6_ films.

## Introduction

Double-perovskite multiferroic La_2_NiMnO_6_ (LNMO) received much attention in recent decades due to its abundant magnetoresistance, magnetocapacitance^1–3^ and dielectric properties^2,4,5^. In particular, the near room temperature magnetic properties have opened up a wealth of promising applications in ferromagnetic semiconductor for spintronics and magnetoelectronics^6–8^. For LNMO materials, chemical ordering of B-site cations (Ni/Mn) strongly affects physical properties, particularly magnetic properties^5,7,9–11^. B-site ordered LNMO shows a single paramagnetic-to-ferromagnetic curie temperature (about 280 K) due to the Ni^2+^-O-Mn^4+^ superexchange interaction^6,12^. In contrast, for B-site disordered LNMO, Ni-O-Ni and Mn-O-Mn bonds are believed to have antiferromagnetic superexchange interactions^6,13,14^. Additionally, ordering of B-site cations affects crystal structure of LNMO. It was reported that orthorhombic LNMO (*Pbnm*) is B-site disordered and monoclinic LNMO (*P*2_1_/*n*) exhibits a long-range B-site ordering^15–18^. For epitaxial films, it is important to note that synthetic conditions such as film-growth temperature and oxygen pressure could affect crystal structure and physical properties of the films^7,19–22^, e.g. BaTiO_3_ films grown on SrTiO_3_ substrates at different oxygen partial pressures^21^. Particularly, B-site ordering in double-perovskite films could be influenced by oxygen partial pressure, e.g. La_2_CoMnO_6_^23^ and La_2_NiMnO_6_^19,24^. It should be noted that the ordering of B-site cations in the double-perovskite films could be improved by controlling the growth temperature, e.g. La_2_CuSnO_6_ films^25^.

In addition, the epitaxial strain of thin films affects the microstructural and physical properties of the films^26–35^. R. J. Zeches *et al*.^28^ reported that strain-driven phase evolution occurs in multiferroic BiFeO_3_ films by controlling the epitaxial strain imposed by the substrate. For the double-perovskite Y_2_NiMnO_6_ films^27^, the magnetic properties and the related ferromagnetic transition temperature (*T*_*c*_) show a strong dependence on the biaxial tensile strain in the films. It is noted that in comparison with the significant studies on the synthesis and properties of bulk materials^1,6,13,18,36,37^, there was a lack of information on the ordering of B-site in double-perovskite films related to the growth parameters and the epitaxial strain induced structure evolution. In our previous work, the short-range ordering of the B-site cation was obtained in the LNMO films coherently grown on the perovskite-type STO and LSAT substrates at 800 °C^26^. Nevertheless, phase stability of LNMO films related to the strain relaxation and the effect of film-growth temperature on the chemical ordering of B-site cations in LNMO films are less concerned.

Nowadays, based on aberration-corrected electron microscopy, advanced imaging techniques in combination with atomic-resolution energy-dispersive X-ray spectroscopy (EDS) mapping provide us with unrivaled capability to understand the structural and chemical properties of materials at the atomic scale^38–44^. In this work, by applying these microscopic analytical techniques, we have systematically investigated phase structure of LNMO films affected by the epitaxial strain and misfit strain relaxation of LNMO films grown on different perovskite-type substrates including KTaO_3_ (KTO), DyScO_3_ (DSO), SrTiO_3_ (STO) and LaAlO_3_ (LAO). We have also studied the effect of film-growth temperature on the ordering of B-site cation in LNMO films. We hope that the present studies would promote the understanding of the effect of the growth process on the structural and physical properties in double-perovskite LNMO films.

## Results and Discussion

It is well known that STO and KTO have a cubic perovskite-type structure with a lattice parameter of 0.3905 and 0.3988 nm at room temperature^45,46^, respectively. In contrast, LAO has a rhombohedrally distorted perovskite-type structure^47^, and DSO has an orthorhombically distorted perovskite-type structure at room temperature^48^. To simplify the discussion, LAO and DSO can be treated as a pseudocubic structure with a lattice parameter (*a*_P_) of 0.3791 nm and 0.3952 (0.3957 nm) nm, respectively, where the subscript P denotes a pseudocubic perovskite-type structure.

**Figure 1:**
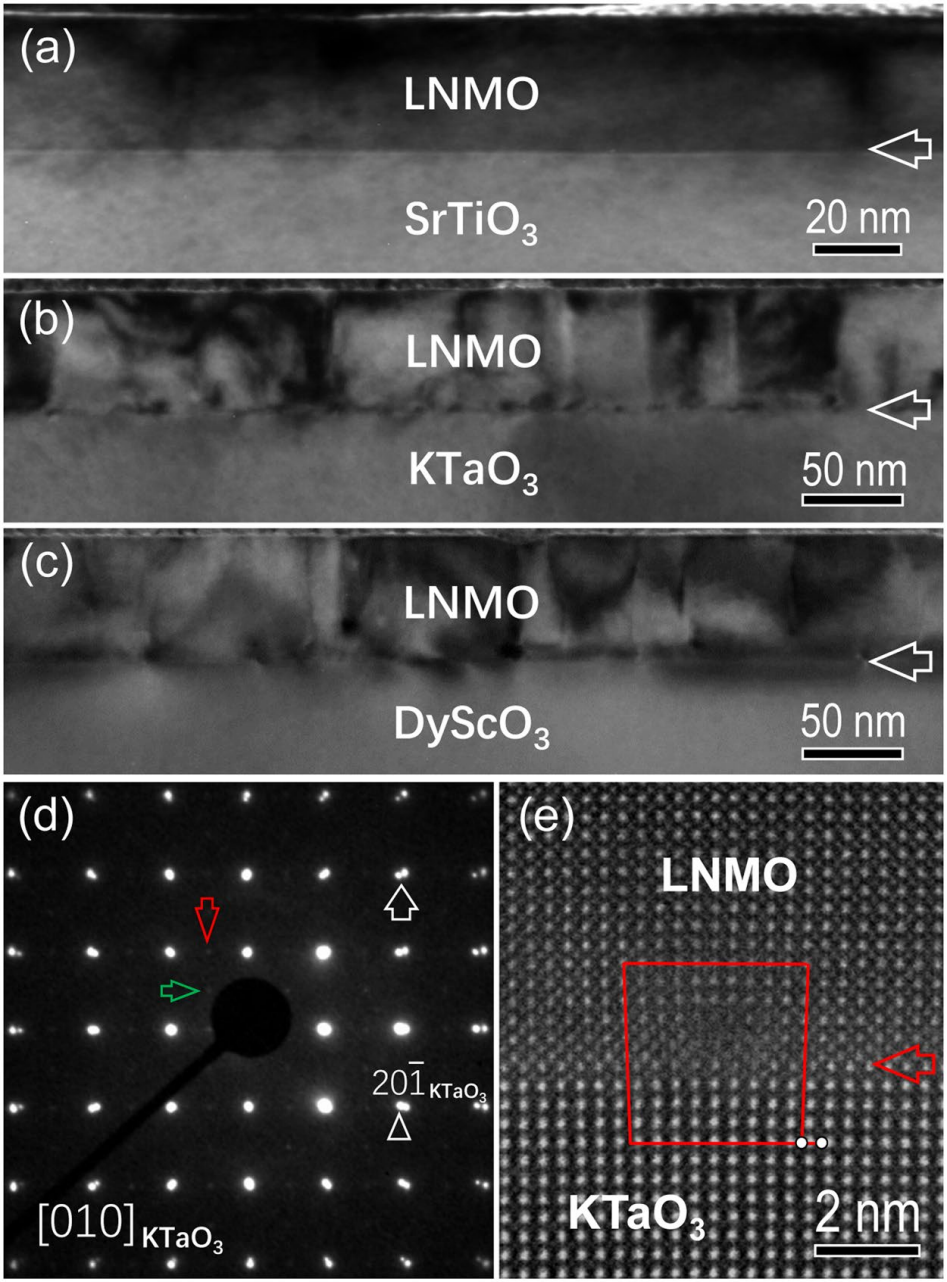
(**a**–**c**) Low-magnification BF-TEM image of the LNMO/STO, LNMO/KTO and LNMO/DSO heterostructures, respectively, viewed along the [010] zone axis of the substrate. The horizontal white arrow denotes the film-substrate interface. (**d**) The SAED pattern from the LNMO/KTO heterostructure, viewed along the [010] KTO zone axis. The splitting of diffraction spots between KTO and LNMO is indicated by a vertical white arrow. The weak diffraction spot from different domains of the LNMO films is denoted by a blue and a red arrow, respectively. (**e**) High-resolution HAADF image showing misfit dislocations at the LNMO/KTO interface. The interface is indicated by a horizontal red arrow.

Figure 1a–c are low-magnification bight-field transmission electron microscopy (BF-TEM) images, showing an overview of LNMO films grown on (001) STO, (001) KTO and (001)_p_ DSO substrates, respectively. The contrast variation is clearly visible in the LNMO films, which allows us to locate the film-substrate interface, as indicated by horizontal white arrows. A typical selected area electron diffraction (SAED) pattern of the LNMO/KTO heterostructure is displayed in Fig. 1d, viewed along the [010] KTO zone axis. A vertical white arrow shows the splitting of diffraction spots between LNMO and KTO, indicating that the relaxation of misfit strain occurs between LNMO film and KTO substrate. In addition, weak diffraction spots from LNMO films but different oriented domains are presented, as denoted by a blue and a red arrow. It should be noted that the weak reflections also appear in the SAED patterns of LNMO film grown on STO and DSO substrates (See Figure S1 of the Supplemental Material). The appearance of weak reflections in the diffraction patterns means that LNMO films have a monoclinic or an orthorhombic structure. It should be mentioned that monoclinic LNMO (*P*2_1_/*n*) and orthorhombic LNMO (*Pbnm*) display structural similarity, which leads to impossible to discern them based on X-ray measurement^17^ and SAED patterns. In contrast to the coherent growth of LNMO films on (001) STO substrate, misfit dislocations are observed at the LNMO/KTO and LNMO/DSO interfaces. Figure 1e shows a typical high-resolution high-angle annular dark field (HAADF) image containing a misfit dislocation at the LNMO/KTO interface, viewed along [010] KTO zone axis. The LNMO/KTO interface is denoted by a horizontal red arrow. It is found that the misfit dislocation has a projected Burgers vector of *a* <100> (*a* is the lattice parameter of KTO), which is obtained by performing a Burgers circuit around the dislocation core, as shown in Fig. 1e.

**Figure 2:**
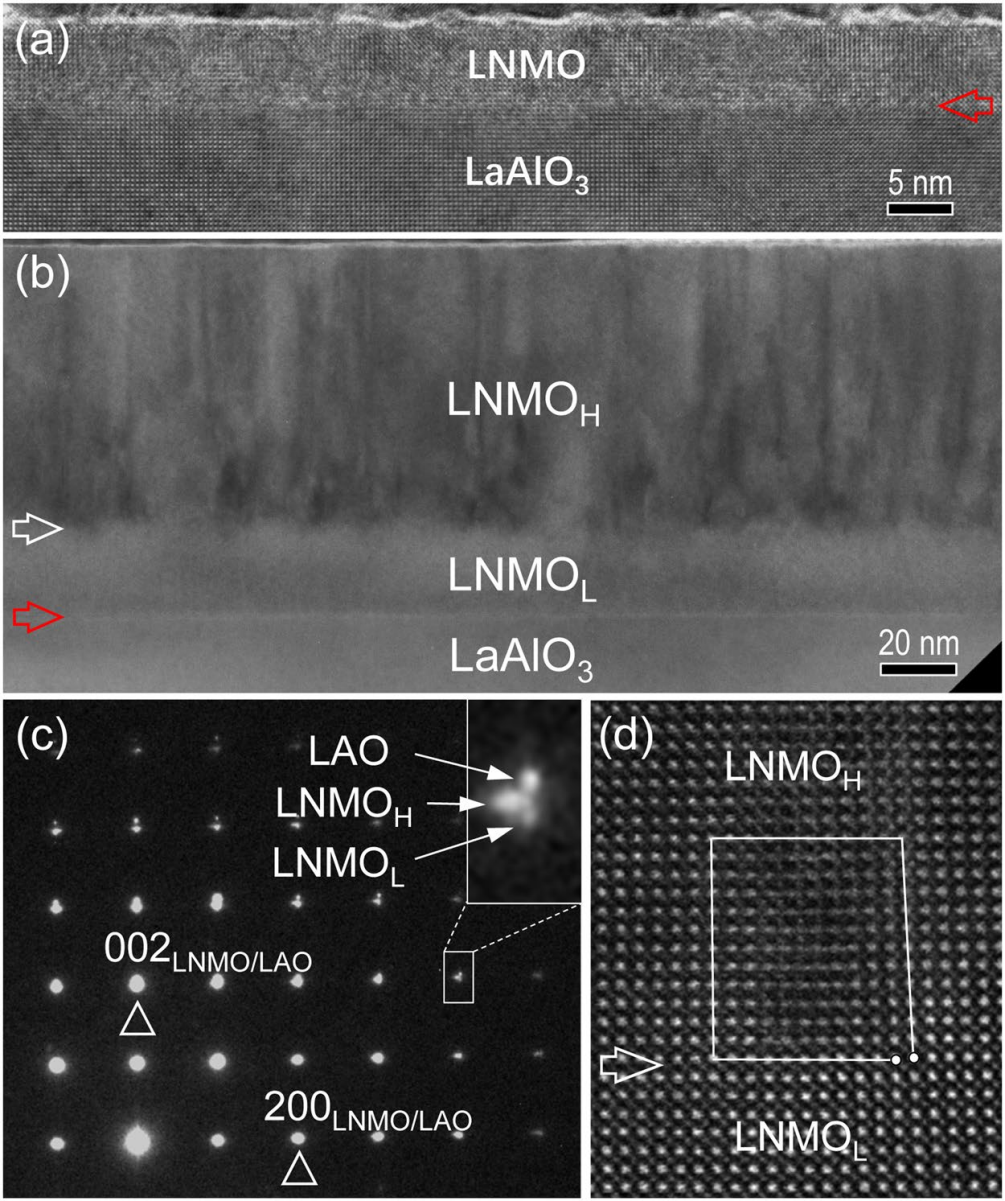
Low-magnification BF-TEM image of the LNMO/LAO heterostructures with 8-nm-thick (**a**) and 100-nm-thick (**b**) LNMO films, respectively. The LNMO/LAO interface is indicated by a horizontal red arrow. The LNMO_L_/LNMO_H_ and LNMO_L_/LAO interface is indicated by a horizontal white and a horizontal red arrow, respectively. (**c**) The SAED pattern recorded in the region of the LAO substrate and 100-nm-thick LNMO films, viewed along the [010]_**p**_ LAO zone-axis. The insert shows the separation of the reflection spot of LNMO_L_, LNMO_H_ and LAO. (**d**) High-resolution HAADF image of misfit dislocations at the LNMO_L_/LNMO_H_ interface. The interface is denoted by a white arrow.

Figure 2a is a low-magnification TEM image of 8-nm-thick LNMO films grown on (001)_**p**_ LAO substrate, viewed along the [010]_**p**_ LAO zone-axis. No misfit dislocations are observed at the LNMO/LAO interface, indicating the LNMO films coherently grow on the LAO substrate. In contrast, for the 100-nm-thick LNMO films grown on (001)_**p**_ LAO substrates, the contrast variation is present within the LNMO films, as shown in a low-magnification BF-TEM image in Fig. 2b. The thick LNMO films exhibit a bilayer structure (referred as LNMO_L_ and LNMO_H_ respectively). The interface between LNMO_L_ and LNMO_H_ is indicated by a horizontal white arrow. The LNMO_L_ layer coherently grows on the LAO substrate with a thickness of about 30 nm. The layer displays a uniform contrast, indicating that it is a monodomain. In comparison, a high density of dark diffuse lines can be seen in the LNMO_H_ layer, starting from the LNMO_L_/LNMO_H_ interface and penetrating in most cases the whole LNMO_H_ layer. Figure 2c shows the SAED pattern of the LNMO/LAO heterostructure. No weak diffraction spots from LNMO_L_ and LNMO_H_ are observed, which indicates that both LNMO layers have a rhombohedral structure. The splitting of diffraction spots can be observed obviously for the high-index reflections. The inset shows the reflection around 402_LAO_ as denoted by a white rectangle in Fig. 2c. It can be seen that there is no in-plane relaxation between LAO and LNMO_L_, which results in that the LNMO_L_ layer has a larger out-of-plane lattice parameter (0.398 ± 0.002 nm). In contrast, in-plane relaxation occurs between LNMO_H_ and LNMO_L_ since the in-plane splitting of reflections can be clearly detected. The epitaxial strain between LNMO_H_ and LNMO_L_ is relaxed by the formation of misfit dislocation at the interface, as shown in Fig. 2d. The projected Burgers vector of the misfit dislocation is *a*_***p***_ <100> (*a*_***p***_ is the lattice parameter of the pseudocubic LNMO), which is obtained by drawing a Burgers circuit around the dislocation core.

It is noted that both LAO and LNMO have a rhombohedral structure in the LNMO/LAO system, which might be in favor of monodomain LNMO films (LNMO_L_) coherently grown on the LAO substrates below a critical thickness. Accordingly, the large compressive strain is accommodated by the elastic deformation of the lattice. Furthermore, with increasing LNMO film thickness above the critical thickness (~30 nm), the film growth mechanism might change from layer-by-layer (Frank-van der Merve mode) growth to island growth to release the large in-plane compressive strain, which results in the formation of the LNMO_H_ layer on the LNMO_L_ layer. Meanwhile, at the LNMO_H_/LNMO_L_ interface misfit dislocations appear due to the coalescence of the LNMO_H_ nuclei during the film growth, which contributes to misfit strain relaxation in the LNMO/LAO system.

**Figure 3:**
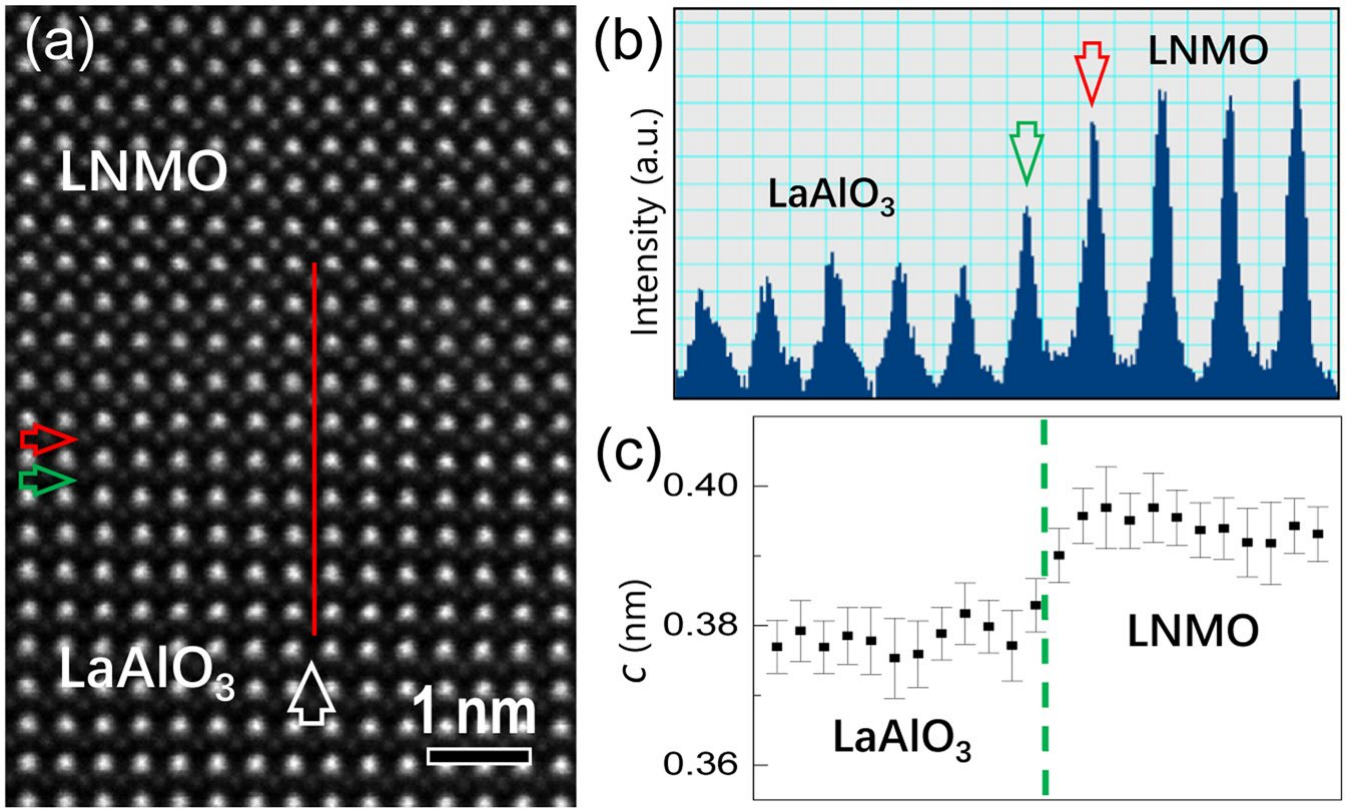
(**a**) Atomic-resolution HAADF image of the LNMO/LAO interface, viewed along the [010]_**p**_ LAO zone axis. The interfacial (Ni, Mn)O_2_ and AlO_2_ layer is denoted by a horizontal red and a horizontal green arrow, respectively. (**b**) The intensity profiles along the red line in Fig. 3a, showing the intensity variation of B-site cation columns in LNMO and LAO across the interface. (**c**) The value of the out-of-plane lattice parameter (*c*) across the LNMO/LAO interface.

To understand the structural properties at the LNMO/LAO interface, high-resolution HAADF investigations of the interface have been performed. A typical atomic-resolution HADDF image of the LNMO/LAO interface is shown in Fig. 3a, viewed along the [010]_p_ LAO zone axis. It is noted that the intensity of atomic columns in the HAADF image is roughly proportional to Z^2^ (where Z is the atomic number averaged in the atomic columns)^49^. The interfacial (Ni, Mn)O_2_ and AlO_2_ layer can be located, as indicated by a horizontal red and a horizontal green arrow, respectively. To obtain the chemical information of B-site cations across LNMO/LAO interface, Fig. 3b displays intensity profiles along the atomic plane marked by a red line in Fig. 3a. Obviously, the intensity of interfacial Al−O columns is higher than that of other Al−O columns, implying that the interface diffusion may occur between Al and Ni/Mn. The out-of-plane lattice parameters in the interface area have been investigated by geometrical lattice mapping directly on the high-resolution HAADF image using a 2D Gaussian fitting approach^50,51^. Figure 3c displays a plot of the out-of-plane lattice spacing against the distance, which provides evidence for the difference in the out-of-plane lattice parameters between LNMO and LAO across the interface.

**Figure 4:**
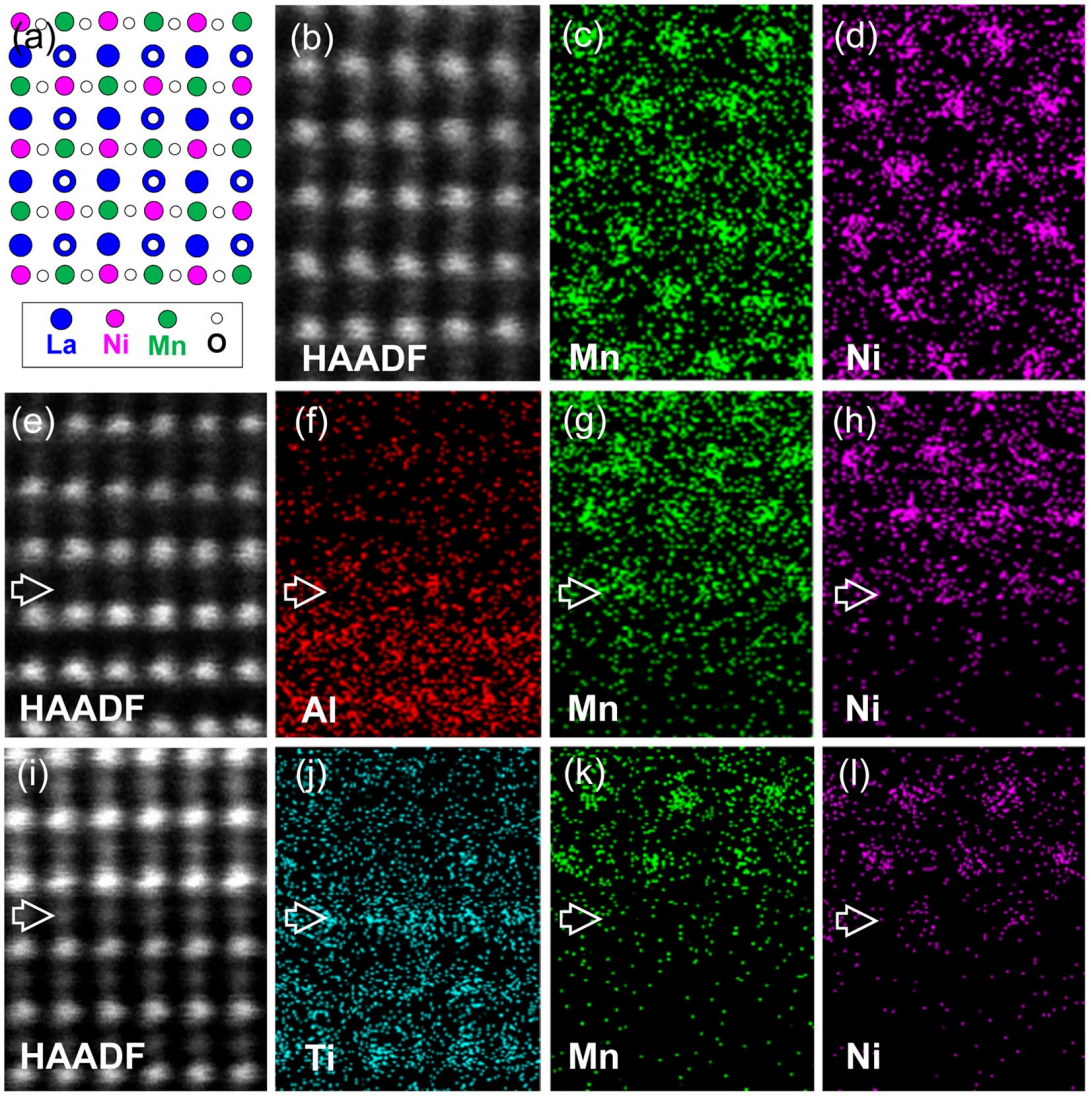
(**a**) Schematic structure model of B-site ordered LNMO, viewed along <110>_**p**_ LNMO zone axis. (**b**–**d**) Atomic-resolution HAADF image of LNMO films and EDS maps of Mn and Ni. (**e**–**h**) HAADF image of the LNMO/LAO interface and EDS maps of Al, Mn and Ni. (**i**–**l**) HAADF image of the LNMO/STO interface and EDS maps of Ti, Mn and Ni.

It is known that B-site ordered LNMO displays ferromagnetic properties due to the Ni-O-Mn interaction. In contrast, apart from Ni-O-Mn superexchange interaction the Mn-O-Mn and Ni-O-Ni antiferromagnetic couplings occur in the short-range B-site ordered LNMO^15^. The ordering of B-site cations (Ni and Mn) in our LNMO films has been investigated by atomic-resolution energy-dispersive X-ray spectroscopy (EDS) mapping. The B-site ordering can be distinguished from the <110>_**P**_ direction of pseudocubic LNMO, as a schematic model illustrated in Fig. 4a. Figure 4b shows a typical high-resolution HAADF image of LNMO films, viewed along the [110]_**p**_ LNMO zone axis. Atomic-resolution EDS mapping for Mn and Ni is displayed in Fig. 4c and d, respectively, which shows a long-range ordering of Ni and Mn in LNMO. It was reported that in contrast to the orthorhombic phase, the monoclinic phase of LNMO is B-site cations ordered^17^. Thus, under our LNMO film-growth conditions, the monoclinic LNMO phase has been obtained. In addition, in contrast to the short-range B-site ordered LNMO films grown at 800 °C (See Figure S2 of the supplemental material), both rhombohedral and monoclinic LNMO films display a long-range B-site ordering at 900 °C (See Figure S3 of the Supplemental Material). It should be mentioned that bond length and bond angle of Ni^2+^-O-Mn^4+^ in the B-site ordered monoclinic and rhombohedral LNMO should be essentially different, which affects the superexchange interactions and the related physical properties (e.g. ferromagnetic) of the films.

In addition, applying atomic-resolution EDS mapping the chemical composition of the LNMO/LAO and LNMO/STO heterointerface has been investigated. Figure 4e shows a high-resolution HAADF image of the LNMO/LAO interface. The corresponding EDS map for Al, Mn and Ni is displayed in Fig. 4f–h, respectively. A monolayer interdiffusion between Mn/Ni and Al was observed at the interface, as indicated by a horizontal white arrow. It can be seen that the rhombohedral LNMO films also exhibit a long-range B-site ordering at the interface area. A high-resolution HAADF image of the LNMO/STO interface is shown in Fig. 4i. The EDS map of B-site cations at the interface is shown in Fig. 4j–l, respectively. The interfacial Ti-O layer is indicated by a horizontal white arrow. Slight interdiffusion between Mn/Ni and Ti occurs at the interface. Also, B-site cations in LNMO display a long-range ordering at the LNMO/STO interface area.

**Figure 5:**
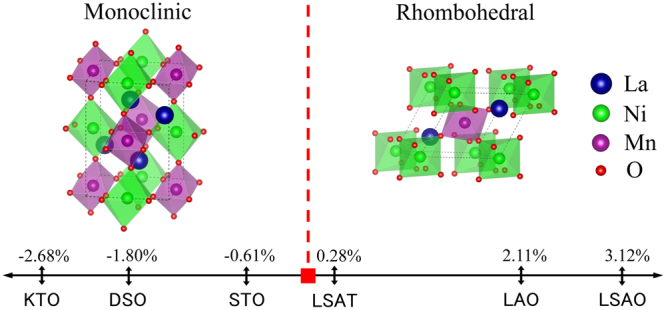
Epitaxial-strain-induced phase transition in LNMO films. The in-plane lattice mismatch values between LNMO and a variety of substrates are given. For monoclinic phase, the value is the minimal in-plane lattice mismatch.

To understand the phase stability of LNMO films, we calculated misfit strain between LNMO films and a variety of substrates, as shown in Table 1. In fact, the unit cell parameters of double-perovskite LNMO structure^52^ can be related to an ideal ABO_3_ perovskite-type unit cell. The monoclinic LNMO has lattice parameters *a* = 5.467 Å, *b* = 5.510 Å (*a*_P1 _≈ *a*_P2_ ≈ $\sqrt{{a}^{2}+{b}^{2}}/2$ ≈ 3.881 Å), *c* = 7.751 Å (*a*_P3_ = *c/2* ≈ 3.875 Å), and *β* = 90.12°, where *a*_P1_, *a*_P2_ and *a*_P3_ are pseudocubic unit-cell lengths. The rhombohedral LNMO has lattice parameters a = 5.475 Å (*a*_P1_ = *a*_P2_ = *a*_P3_ ≈ $a/\sqrt{2}$≈ 3.871 Å), and *β* = 60.67°. In the case of the monoclinic LNMO on the substrates, several types of film-substrate orientation relationship may exist depending on the crystallographic structure of the substrate, e.g. O_1_, O_2_ and O_3_ in the LNMO/DSO system, which leads to different in-plane strains in the two orthogonal directions. Also, anisotropic in-plane strain may occur in the rhombohedral LNMO because of the substrate with different in-plane lattice parameters, e.g. in the LNMO/DSO system. As list in the Table 1, both monoclinic and rhombohedral LNMO films undergo tensile strain if the films coherently grow on STO, DSO and KTO. Nevertheless, in-plane mismatch strain of monoclinic LNMO is smaller than that of rhombohedral LNMO. In contrast, although compressive epitaxial strain would be induced in both monoclinic and rhombohedral LNMO films if the films coherently grow on LAO, (001) (La, Sr)(Al,Ta)O_3_ (LSAT) (*Pn* $\overline{3}$ *m*)^53^ and (001) LaSrAlO_4_ (LSAO) (*I*4*/mmm*)^54^, in-plane mismatch strain of monoclinic LNMO is larger than that of rhombohedral LNMO. The different in-plane mismatch strain possibly results in the formation of monoclinic LNMO films under the tensile strain and rhombohedral LNMO films under the compressive strain. The relationship of strain-induced phase transition in LNMO films is shown in Fig. 5.

**Table 1:** Crystal structure and lattice parameter of STO (ref.^45^), KTO (ref.^46^), LAO (ref.^47^), DSO (ref.^48^), LSAT (ref.^53^), and LSAO (ref.^54^) substrates, and the in-plane lattice mismatch between LNMO (ref.^52^) and various substrates, which is calculated by using the equation of Δ*f* = (*a*_f_ − *a*_0_)/*a*_0_ * 100% (*a*_0_ and *a*_f_ are the lattice parameter of substrate and pseudocubic LNMO, respectively).

Materials	Crystal structure	Unit cell lattice parameters (Å)			Pseudocubic lattice parameters (Å)			In-plane lattice mismatch (with Monoclinic LNMO)		In-plane lattice mismatch (with Rhombohedral LNMO)	
		*a*	*b*	*c*	*a*_*P1*_	*a*_*P2*_	*a*_*P3*_	*Δf*_*M1*_	*Δf*_*M2*_	*Δf*_*R1*_	*Δf*_*R2*_
LNMO	*R* $\overline{3}$	5.475	5.475	5.475	3.871	3.871	3.871				
LNMO	*P*2_1_/*n*	5.467	5.510	7.751	3.881	3.881	3.875				
KTO	*Pm* $\overline{3}$ *m*	3.988	3.988	3.988				−2.68%	−2.83%	−2.93%	−2.93%
DSO	*Pbnm*	5.449	5.726	7.913	3.952	3.952	3.957	−1.80% (O_1_) −1.92% (O_2_) −1.80% (O_3_)	−1.92% (O_1_) −1.95% (O_2_) −2.07% (O_3_)	−2.05%	−2.17%
STO	*Pm* $\overline{3}$ *m*	3.905	3.905	3.905				−0.61%	−0.77%	−0.87%	−0.87%
LSAT	*Pn* $\overline{3}$ *m*	7.720	7.720	7.720	3.860	3.860	3.860	0.54%	0.39%	0.28%	0.28%
LAO	*R* $\overline{3}$	5.360	5.360	13.086	3.791	3.791	3.791	2.37%	2.22%	2.11%	2.11%
LSAO	*I*4/*mmm*	3.754	3.754	12.649				3.38%	3.22%	3.12%	3.12%

According to our experimental results, the small lattice mismatch could be fully accommodated by the elastic deformation of the lattice and oriented domains, which leads to the coherent growth of films on substrates, e.g. in the LNMO/STO and LNMO/LSAT systems^26^. In most cases, with the increase of the film-substrate lattice mismatch, apart from the formation of oriented domains in the LNMO films misfit dislocations appear at the film-substrate interface, contributing to misfit strain relaxation. As a result, the films and the substrates form the semi-coherent interface, e.g. in the LNMO/DSO and LNMO/KTO systems. It is important to note that both coherently and semi-coherently strained LNMO films under the tensile strain exhibit a monoclinic structure and under the compressive strain a rhombohedral structure.

Additionally, in comparison with the partially disordered LNMO films grown at 800 °C^26^, the LNMO films prepared at 900 °C exhibit a long-range B-site ordering. In fact, with the exception of the growth temperature, the deposition conditions were held the same for preparing LNMO films in these two works. In other words, our works indicate that B-site ordering in LNMO films could be improved efficiently by increasing growth temperature. It is noted that the growth temperature can significantly influence the film growth kinetics, e.g. the thermal diffusion of the cations in the lattice^55–57^. Increasing film-growth temperature increases the kinetic energy of the diffusing cations in the LNMO, which could lead the B-site cations (Ni and Mn) to arrange themselves in the cation sublattice in an ordered manner. In fact, the ordering of B-site in bulk LNMO could be improved by changing the thermal annealing parameters^24^. In this viewpoint, post-growth annealing of the LNMO films may provide an alternative way of manipulating the B-site ordering in order to enhance their performance in device applications.

## Conclusions

In summary, by means of advanced electron microscopy we have demonstrated that the misfit strain of LNMO films grown on different substrates could be partially released by the formation of oriented domains within the films and misfit dislocations at the interfaces. Importantly, phase stability of LNMO films could be tuned effectively by elastic strains imposed by the substrate. Under tensile stain, LNMO films exhibit a monoclinic structure, while under compressive strain LNMO films are rhombohedral. In addition, a long-range B-site ordered LNMO films have been successfully prepared at 900 °C. Our work indicates that the ordering of B-site cations in LNMO films could be affected by controlling film-growth temperature. Importantly, the atomic-scale characterization of chemical and structural properties of double-perovskite multiferroic LNMO films would extend our understanding of the process-structure-property relationship in the materials and explore their potential applications.

## Methods

LNMO thin films were deposited on STO, KTO, DSO and LAO substrates by the pulsed laser deposition (PLD) technique. A KrF excimer laser (wavelength: 248 nm) was applied for ablation of a sintered LNMO target. The film deposition conditions were held the same for all different substrates, including the substrate temperature (900 °C), oxygen pressure (250 mTorr), target-substrate distance (10 cm), laser fluence (2 J/cm^2^), and repetition frequency (5 Hz).

Cross-sectional TEM specimens were prepared by conventional TEM sample preparation technique and focused ion beam (FIB) lift-out technique using an FEI Helios600i FIB/SEM system^58^. BF-TEM images and SAED patterns were acquired on a JEM 2100 microscope. Atomic-resolution HAADF imaging and EDS mapping were performed on a JEOL ARM200F microscope equipped with an aberration corrector for a probe*-*forming system and an Oxford X-MaxN 100TLE spectrometer, operated at 200 kV. The HAADF detector covered an angular range of 90–176 mrad and the convergence semi-angle was set to 22 mrad for HAADF imaging.

## Electronic supplementary material

Supplemental Information
