# Supplementary material for: B-site ordering and strain-induced phase transition in double-perovskite La2NiMnO6 films
Source: Sci Rep. 2018 Feb 6;8:2516. doi: 10.1038/s41598-018-20812-4 (PMC5802844; doi:10.1038/s41598-018-20812-4)
Supplement: Supplementary file 1 — Supplemental Information [file 41598_2018_20812_MOESM1_ESM.doc]

**Supplemental Materials**

**B-site ordering and strain-induced phase transition in double-perovskite La_2_NiMnO_6_ films**

Sheng-Qiang Wu^1^, Sheng Cheng^2^, Lu Lu^2^, Ming Liu^2^, Xiao-Wei Jin^2^, Shao-Dong Cheng^1,2^, Shao-Bo Mi1,[[1]](#footnote-2)*

^1^*State Key Laboratory for Mechanical Behavior of Materials, Xi'an Jiaotong University, Xi'an 710049, China*

*^2^School of Microelectronics, Xi'an Jiaotong University, Xi'an 710049, China*


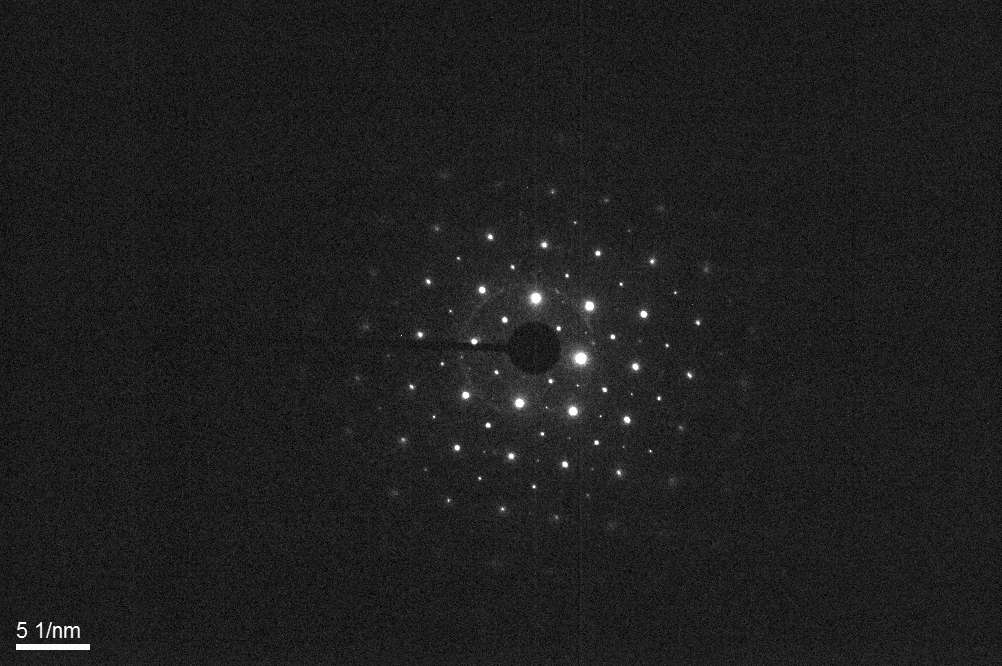

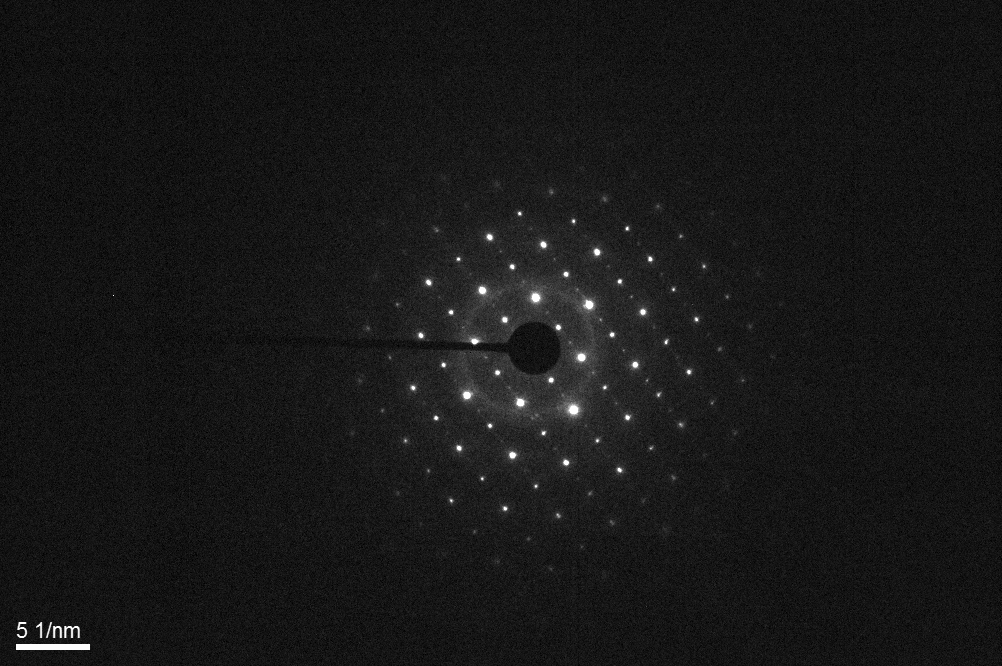

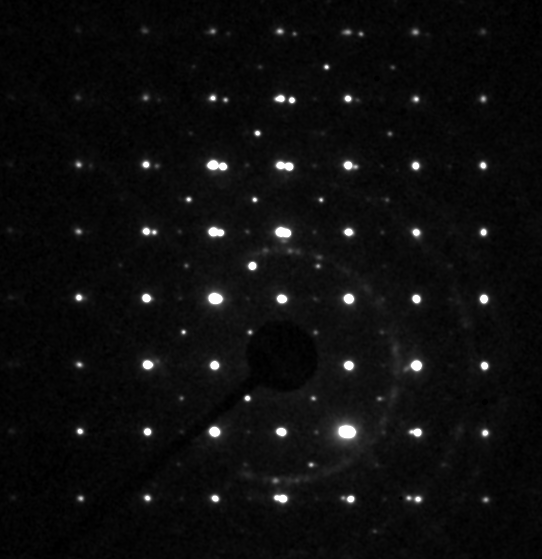


[001]_p_DSO

[001]STO

[001]STO

(a)

(b)

(c)

**Figure S1. Typical selected-area electron diffraction (SAED) patterns of the LNMO/STO (a, b) and LNMO/DSO (c) heterostructures, viewed along the [001] STO and [001]_p_ DSO zone axis, respectively.** Weak diffraction spots from LNMO films are presented in (a)-(c), as denoted by red arrows, indicating that LNMO films have a monoclinic or an orthorhombic structure on STO and DSO. In addition, no splitting of diffraction spots between LNMO and STO is visible in (a) and (b). In contrast, splitting of diffraction spots between LNMO and DSO can be observed, as indicated by a vertical white arrow in (c).

**(a)**

**(b)**


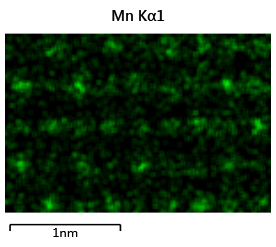

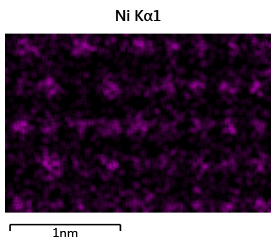

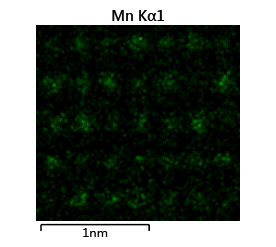

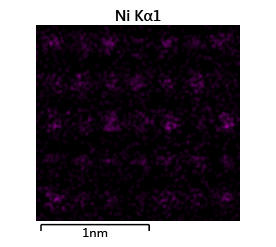


**(c)**

**(d)**

**Figure S2. Atomic-resolved EDS maps of rhombohedral and monoclinic LNMO films showing short-range B-site ordered films prepared at 800 °C.** (a) and (b) EDS maps of Mn and Ni in monoclinic LNMO films grown on STO substrates. (c) and (d) EDS maps of Mn and Ni in rhombohedral LNMO grown on LSAT substrates. The vertical white arrows demonstrate the disordered B-site atomic columns in LNMO.

**Figure S3. Atomic-resolved EDS maps of rhombohedral and monoclinic LNMO films showing long-range B-site ordered films prepared at 900 °C.** (a) and (b) EDS maps of Mn and Ni in rhombohedral LNMO films grown on LAO substrates. (c) and (d) EDS maps of Mn and Ni in monoclinic LNMO grown on STO substrates.


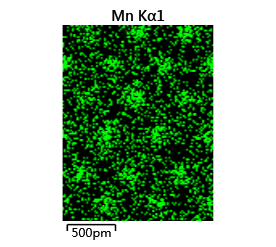

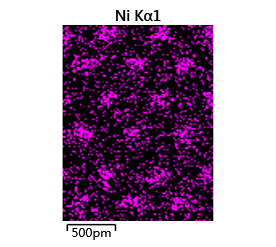


**(a)**

**(b)**

**(c)**

**(d)**


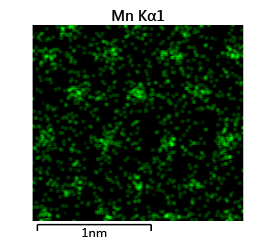

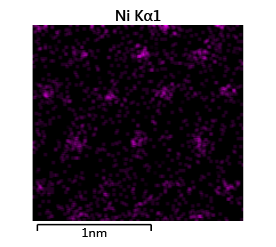


1. ^^ Corresponding author:

   [shaobo.mi@xjtu.edu.cn](mailto:shaobo.mi@xjtu.edu.cn) (S.-B. Mi) [↑](#footnote-ref-2)
